# Meal Regularity Plays a Role in Shaping the Saliva Microbiota

**DOI:** 10.3389/fmicb.2020.00757

**Published:** 2020-04-24

**Authors:** Jannina Viljakainen, Sajan C. Raju, Heli Viljakainen, Rejane Augusta de Oliveira Figueiredo, Eva Roos, Elisabete Weiderpass, Trine B. Rounge

**Affiliations:** ^1^Folkhälsan Research Center, Helsinki, Finland; ^2^Faculty of Medicine, University of Helsinki, Helsinki, Finland; ^3^Department of Food and Nutrition, University of Helsinki, Helsinki, Finland; ^4^International Agency for Research on Cancer – World Health Organization, Lyon, France; ^5^Department of Research, Cancer Registry of Norway, Oslo, Norway; ^6^Department of Informatics, University of Oslo, Oslo, Norway

**Keywords:** eating habits, saliva, human microbiota, breakfast, dinner, adolescent

## Abstract

**Background:**

Diet may influence health directly or indirectly via the human microbiota, emphasizing the need to unravel these complex relationships for future health benefits. Associations between eating habits and gut microbiota have been shown, but less is known about the association between eating habits and saliva microbiota.

**Objective:**

The aim of this study was to investigate if eating habits and meal patterns are associated with the saliva microbiota.

**Methods:**

In total, 842 adolescents, aged 11–14 years, from the Finnish Health in Teens (Fin-HIT) study cohort were included in this study. Eating habits and breakfast and dinner patterns were derived from a web-based questionnaire answered in school. Three major eating habit groups were identified: fruit and vegetable avoiders (FV avoiders), healthy and unhealthy. Microbiota profiles were produced from 16S rRNA gene (V3–V4) sequencing of DNA from the saliva samples. Statistical models were adjusted for gender, age, parental language, body mass index (BMI) categories, and sequencing depth.

**Results:**

Regular breakfast eaters had a higher alpha diversity (Shannon index with mean (standard error of means) 2.27 (0.03) vs. 2.22 (0.03), *p* = 0.06, inverse Simpson’s index with 6.27 (0.17) vs. 5.80 (0.02), *p* = 0.01), and slight differences in bacterial composition (PERMANOVA: *p* = 0.001) compared with irregular breakfast eaters. A similar trend in alpha diversity was observed between regular and irregular dinner eaters (Shannon index with 2.27 (0.03) vs. 2.22 (0.03), *p* = 0.054, inverse Simpson’s index with 6.23 (0.17) vs. 6.04 (0.22), *p* = 0.28), while no difference was found in composition (PERMANOVA: *p* = 0.08). No differences were identified between eating habit groups and saliva microbiota diversity (Shannon index *p* = 0.77, inverse Simpson’s index *p* = 0.94) or composition (PERMANOVA: *p* = 0.13). FV avoiders, irregular breakfast eaters and irregular dinner eaters had high abundances of *Prevotella*.

**Conclusion:**

Regularity of eating, especially breakfast eating, was associated with more diverse saliva microbiota and different composition compared with irregular eaters. However, the dissimilarities in composition were small between regular and irregular breakfast eaters. Our results suggest that *Prevotella* abundances in saliva were common in FV avoiders and meal skippers. However, the clinical implications of these findings need to be evaluated in future studies.

## Introduction

A healthy diet maintains well-being (Skerrett and Willett, 2010) and contributes to the human microbiota composition (Wu et al., 2011). There is a growing body of research on the association between eating habits and gut microbiota (Santacruz et al., 2009; De Filippo et al., 2010; Wu et al., 2011; Cotillard et al., 2013), whereas only a few studies have explored the relationship between eating habits and the saliva microbiota (De Filippis et al., 2014; Ercolini et al., 2015; Hansen et al., 2018). The saliva microbiota is considered relatively stable and resilient and may reflect health status in individuals (Stahringer et al., 2012).

Studies on associations between eating habits and the saliva microbiota vary in design and sample size and show conflicting results (De Filippis et al., 2014; Ercolini et al., 2015; Hansen et al., 2018). De Filippis et al. (2014) showed no differences in saliva microbiota diversity and composition between Italian adults aged 18–55 years following omnivore (*n* = 55), ovo-lacto vegetarian (*n* = 55) and vegan (*n* = 51) diets for a minimum of 1 year (De Filippis et al., 2014). On the contrary, the composition of saliva microbiota differed between Danish vegans (*n* = 78) and omnivores (*n* = 82) who followed their diets for at least 1 year in a more recent study of adults aged 18–65 years (Hansen et al., 2018): the vegans had a higher abundance of *Neisseria* in the saliva compared with the omnivores. In children with celiac disease, the effect of a 60-day dietary treatment on saliva microbiota was evaluated (Ercolini et al., 2015); this study showed that dietary treatment of 14 African celiac children with an Italian-style gluten-free diet (a typical Western omnivore diet) resulted in reduced diversity and alterations in the microbiota composition.

Of note, research on gut microbiota and eating habits in children is limited (De Filippo et al., 2017; Shankar et al., 2017). One of these studies identified differences in gut microbiota between the Western (*n* = 14) and Mediterranean diets (*n* = 28) (Shankar et al., 2017). The Mediterranean diet, containing high amounts of fiber, was associated with a high abundance of *Prevotella*, while the Western diet, characterized by high amounts of animal protein and saturated fat, was associated with a high abundance of *Bacteroides* (Shankar et al., 2017). They suggest that the type of diet consumed may shape the intestinal microbiota (Shankar et al., 2017). There is some evidence of a relationship between the Western diet and dysbiosis, including reduced gut microbiota diversity (Chiba et al., 2019). However, these aforementioned studies on children involve small sample sizes, ranging from 29 to 42 children in total (De Filippo et al., 2017; Shankar et al., 2017), suggesting insufficient statistical power according to Sze and Schloss (2016).

The timing of eating has also been proposed to affect the human microbiota (Collado et al., 2018). To our knowledge, there is only one study on the timing of eating and saliva microbiota, healthy subjects (*n* = 10) (Collado et al., 2018). This cross-over, randomized intervention study (Collado et al., 2018) showed that diversity was higher in late eaters at two time points compared with early eaters (Collado et al., 2018). Comparable studies on the timing of eating and gut microbiota are also scarce. A study (Kaczmarek et al., 2017) demonstrated that eating behavior (including the timing of eating) affects both the gut microbiota composition and function in 28 healthy men and women throughout the day.

Healthy eating, such as a regular meal pattern, has been suggested to associate with lower BMI in Finnish children (Lehto et al., 2011) and protects from overweight and obesity in Finnish adolescents (Jääskeläinen et al., 2013). Whereas, unhealthy eating habits, including avoiding fruits and vegetables, have been associated with underweight in Finnish adolescents (Viljakainen et al., 2019). In particular, unhealthy eating habits are a concern regarding their effect on unfavorable health outcomes. However, little is known about the possible association between eating habits and saliva microbiota at any age. Thus, we examine whether eating habits and meal patterns are associated with saliva microbiota in Finnish adolescents utilizing microbiota profiles from 842 previously sequenced saliva samples (Raju et al., 2019). Earlier, we have identified three different eating habit groups in the same cohort we are presenting here: “fruit and vegetable avoiders,” “healthy,” and “unhealthy,” which are described elsewhere in detail (de Oliveira Figueiredo et al., 2019). We hypothesize that these eating habits and meal patterns are associated with microbial diversity and composition in saliva in a presumably healthy population.

## Materials and Methods

This cross-sectional study includes 842 adolescents aged 11–14 years from the Finnish Health in Teens (Fin-HIT) study cohort, described in detail elsewhere (Figueiredo et al., 2019). Briefly, altogether 11,407 adolescents participated in the Fin-HIT study in Southern, Middle and Northern Finland between the years 2011 and 2014 (Figueiredo et al., 2019). From the Fin-HIT cohort, 1,000 adolescents were randomly selected for the microbial analyses. We excluded 25 adolescents with consent withdrawals, 21 without BMI measurement information, 21 using antibiotics within 3 months prior to sampling and 91 samples with low sequence depth (<500 read pairs).

Adolescents filled in a web-based baseline questionnaire, including eating habits and breakfast and dinner patterns on an electronic tablet in school (de Oliveira Figueiredo et al., 2019; Figueiredo et al., 2019; Viljakainen et al., 2019). In addition, participants provided an unstimulated saliva sample, and trained fieldworkers measured their height and weight in a standardized way (Figueiredo et al., 2019) which we used to calculate body mass index (BMI) (kg/m^2^), described in detail elsewhere (Sarkkola et al., 2016). We used age- and gender-specific International Obesity Task Force (IOTF) cut-offs to categorize BMI as underweight, normal-weight and overweight or obese (Cole and Lobstein, 2012).

The adolescents and their parents provided informed written consent. We recorded adolescents’ gender, age and parental language from the consent form or questionnaires, and then linked them to the National Population System at the Population Register Center. The Coordinating Ethics Committee of the Hospital District of Helsinki and Uusimaa (169/13/03/00/10) approved the study protocol.

### Saliva Sample

Adolescents provided saliva samples mostly between breakfast and lunch on school days. We used the Oragene-DNA (OG-500) Self-Collection Kit (DNA Genotek Inc., Ottawa, ON, Canada) for collecting the saliva samples and stored them at room temperature. DNA was extracted with intensive lysis including lysozyme, mutanolysin and lysostaphin, bead-beating for 1 min and automated extraction using a Chemagic protocol (PerkinElmer, United States). DNA was included in equal volumes in the PCR amplifications. The protocol is described in detail by Raju et al. (2018).

### Eating Habits

The frequency of food consumption was obtained with a 14-item food frequency questionnaire according to the past month, as previously described (de Oliveira Figueiredo et al., 2019). Adolescents reported their food consumption, which was evaluated by a 7-point scale varying from 0 (not consumed) to 6 (consumed several times per day). In this cohort, we have previously reported three main eating habit groups, which were derived by using K-means cluster analysis. Factor analysis provided five factors from ten food items, and these factors were used in K-means cluster analysis to identify the three eating habits: “fruit and vegetable avoiders (FV avoiders),” “healthy,” and “unhealthy” (de Oliveira Figueiredo et al., 2019), which are illustrated in Supplementary Figure S1. We considered adolescents as FV avoiders when they consumed fewer fresh vegetables, fruits, and berries compared with the others. Healthy eaters most frequently ate dark bread, fresh vegetables, fruits, and berries. While unhealthy eaters had the highest intake of sweet pastries, sugary juice drinks, fast food (hamburgers or hot dogs), and salty snacks. All eating habit groups included indicatory food items of healthy and unhealthy diets, which were recommended previously by the Health Behavior in School-aged Children study protocol (Currie et al., 2004, 2010)

Breakfast and dinner patterns were considered regular when eaten every school day, otherwise it was considered irregular (de Oliveira Figueiredo et al., 2019; Viljakainen et al., 2019). The cohort lacks information on timing of meals (on the day of sample donation), portion size and data for weekend. In Finland, the majority of children eat a free warm lunch in school 5 days a week, thus, lunch consumption was not included in our analyses.

### 16S rRNA Gene Amplification and Sequencing

The 16S rRNA gene amplification and sequencing protocol, including assessment of repeatability and contamination, has been described in detail elsewhere (Raju et al., 2018). In summary, we used the TruSeq (TS)-tailed 1-step amplification protocol amplifying the 16S rRNA gene V3-V4 region with the primers S-D-Bact-0341-b-S-17 (5′-CCTACGGGNGGCWGCAG-3′) and S-D-Bact-0785-a-A-21 (5′-GACTACHVGGGTATCTAATCC-3′) (Klindworth et al., 2013). The libraries were sequenced on the Illumina HiSeq1500 platform (Illumina, Inc., San Diego, CA, United States) with 271 × 2 bp paired end to provide read overlap and high-quality assembly (Raju et al., 2018). This approach provided high reproducibility and low contamination (Raju et al., 2018). The total number of all assembled read pairs was 133 million. The average per sample was 1.6 million pairs and the median was 118,641 pairs. We removed 91 samples due to too few pairs in the subsampling.

### Operational Taxonomic Units (OTUs)

The sequencing data was processed into OTUs using Mothur (Version v.1.35.1) following the MiSeq standard operational procedure (Schloss et al., 2009), which are described in detail by Raju et al. (2018). In summary, the sequences were filtered on quality and the read pairs were assembled. The assembled reads were aligned to the SILVA 16S rRNA gene database (Version V119) and clustered into OTUs with a >98% similarity cut-off to enable species’ resolution. In total 6536 OTUs were identified, whereas approximately 1000 of the most abundant OTUs were included in the statistical analyses, depending on the specific sample set. The data were normalized with a subsampling threshold of 500. Samples below this threshold were excluded from the analyses. We rarefied data to normalize the sequenced depth variability. After rarefaction, we calculated the Shannon index to assess richness (Wagner et al., 2018) and inverse Simpson’s index to assess richness and evenness of the microbiota for alpha diversity (Simpson, 1949). The Bray–Curtis dissimilarity index (calculated from counts) was used for assessing compositional differences of the saliva microbiota between eating habits and breakfast and dinner patterns. The Bray–Curtis index of 0 means that two samples have the same composition, while the Bray–Curtis index of 1 means that two samples do not share any species together. Data for the abundance analyses were pre-filtered to include the OTUs with a minimum of 20 sequences.

### Statistical Analysis

The Shapirov–Wilk test was used for normality test for alpha diversity. *T*-tests assessed alpha diversity between breakfast and dinner patterns. Alpha diversity was also compared between eating habits using analysis of variance (ANOVA) without adjustments. For this test, the homogeneity of variances was checked using Levene’s test. Alpha diversity was then compared between eating habits and breakfast and dinner patterns using analysis of covariance (ANCOVA). All analyses evaluating eating habit and breakfast and dinner patterns were adjusted for gender, age, parental language, BMI categories and sequencing depth (full adjustment). We adjusted for parental language which may be used as a proxy for socioeconomic status (Härtull, 2018) and different haplotypes (Palo et al., 2008). We adjusted for gender (Raju et al., 2019), age (Yatsunenko et al., 2012; Lif Holgerson et al., 2015), and BMI (Piombino et al., 2014), which have found to associate with oral and saliva microbiota. Furthermore, we adjusted for sequencing depth since it affects the data quality even after rarefaction. Additionally, we adjusted for eating habits when assessing breakfast and dinner patters. Interactions between gender and each of the dietary variables (eating habits and breakfast and dinner patterns) were tested.

The comparisons of composition between eating habits and breakfast and dinner patterns were conducted by a permutational analysis of variance (PERMANOVA) for the matrix of Bray–Curtis index and illustrated by Principal Coordinates Analysis (PCoA) plots. For the analyses, we used adonis and betadisper functions in Phyloseq and Vegan R-packages. The homogeneity of multivariate dispersion between groups was tested to check the assumptions for the model. Variability homogeneity was found between eating habit groups and between dinner pattern groups. Since there was heterogeneity between regular and irregular breakfast pattern groups, we used a random sampling of adolescents who regularly ate breakfast to obtain balanced groups, since PERMANOVA is robust for a balanced design (Anderson and Walsh, 2013; Odintsova et al., 2017). We selected 142 adolescents with a regular breakfast pattern and compared those with the 142 adolescents with the irregular breakfast pattern to balance the number of participants. These analyses were repeated 30 times to confirm the results obtained with PERMANOVA.

We carried out sensitivity analyses for alpha and beta diversities in: (1) regular breakfast eaters’ subgroup for eating habits (*n* = 700, 83%), (2) regular dinner eaters’ subgroup for eating habits (*n* = 694, 82%), (3) healthy eaters’ subgroup for breakfast and dinner eaters (*n* = 383, 46%), and (4) FV avoiders’ subgroup for breakfast and dinner eaters (*n* = 361, 43%).

The DESeq2 R-package (Version 3.2.4) tested the differential abundance of OTUs using a negative binomial model. In addition, the multiple comparison method was used to compare between two of the three eating habit groups: FV avoiders, healthy and unhealthy. We also tested the differential abundances of OTUs between irregular breakfast vs. regular breakfast patterns and irregular dinner vs. regular dinner patterns. We used false discovery rate (FDR) adjustment for *p*-value. All analyses were performed in R and SPSS version 24.0 (IBM Corp., Armonk, NY, United States).

We illustrated the differential abundances OTUs with volcano plots for: (a) FV avoiders vs. healthy, (b) unhealthy vs. healthy, (c) FV avoiders vs. unhealthy, (d) irregular breakfast vs. regular breakfast patterns, and (e) irregular dinner vs. regular dinner patterns.

## Results

### Background Characteristics

We describe in detail adolescents’ characteristics, such as gender, age and parental language in Table 1. In total, 842 participants were grouped into three different eating habits: FV avoiders (42.9%), healthy (45.5%), and unhealthy (11.6%) eaters. Most of the adolescents followed regular breakfast (83.1%) and dinner patterns (82.4%).

**TABLE 1:** Characteristics of the adolescents.

		Total (*N* = 842)	
		n	%
Eating habits	Fruit and vegetable avoider	361	42.9
	Healthy	383	45.5
	Unhealthy	98	11.6
Breakfast patterns	Irregular	142	16.9
	Regular	700	83.1
Dinner patterns	Irregular	148	17.6
	Regular	694	82.4
Gender	Boy	387	46.0
	Girl	455	54.0
Age		11.7^a^	0.37^b^
Parental language	Finnish	719	85.4
	Swedish	89	10.6
	Other	34	4.0
BMI categories	Underweight	116	13.8
	Normal-weight	617	73.3
	Overweight and obese	109	12.9
Alpha diversity	Shannon diversity index	2.26^a^	±0.29^b^
	Inverse Simpson’s index	6.12^a^	±1.94^b^

*^a^Mean. ^b^Standard deviation. BMI, Body Mass Index.*

### Eating Habits and Saliva Microbiota

**FIGURE 1:**
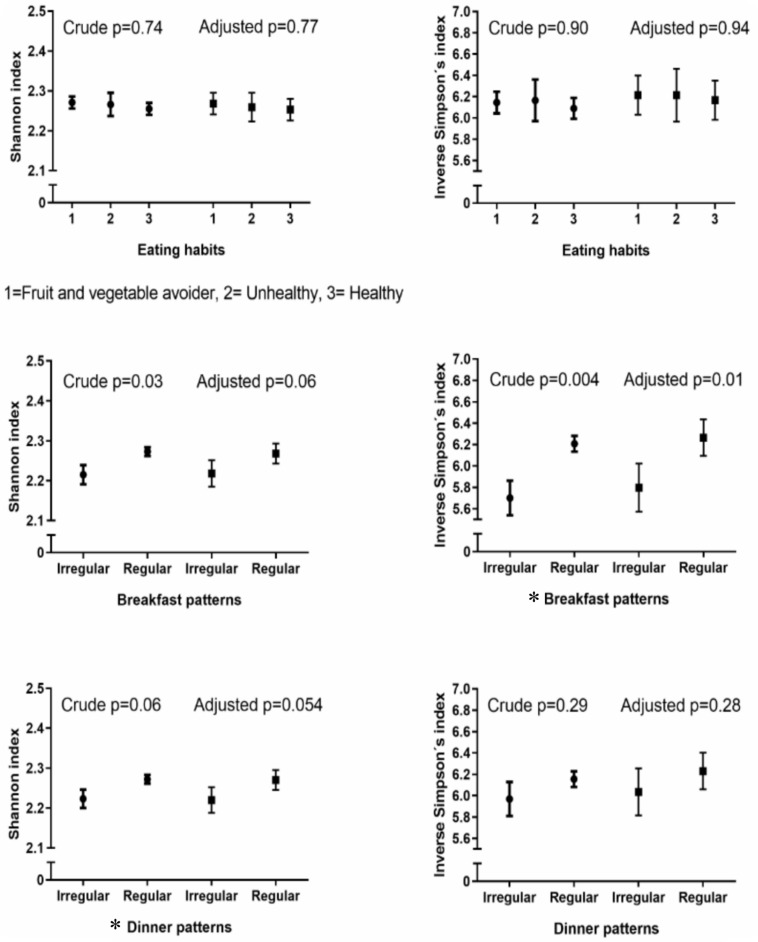
Crude and adjusted alpha diversity with eating habits and breakfast and dinner patterns. Crude *p*-values for breakfast and dinner patterns were calculated with *t*-tests and crude *p*-values for eating habits with analysis of variance (ANOVA). All the adjusted *p*-values were calculated with Analysis of Covariance (ANCOVA). Adjusted for: gender, age, parental language, body mass index (BMI) categories and sequencing depth. ^∗^Statistically significant.

Alpha diversity, evaluated by the Shannon diversity index and inverse Simpson’s index, did not differ between eating habits (ANCOVA: *p* = 0.77 and *p* = 0.94, respectively) (Figure 1). There was no interaction between gender and eating habits with regards to alpha diversity (*p* = 0.18 for the Shannon diversity and *p* = 0.21 for inverse Simpson’s indices).

**FIGURE 2:**
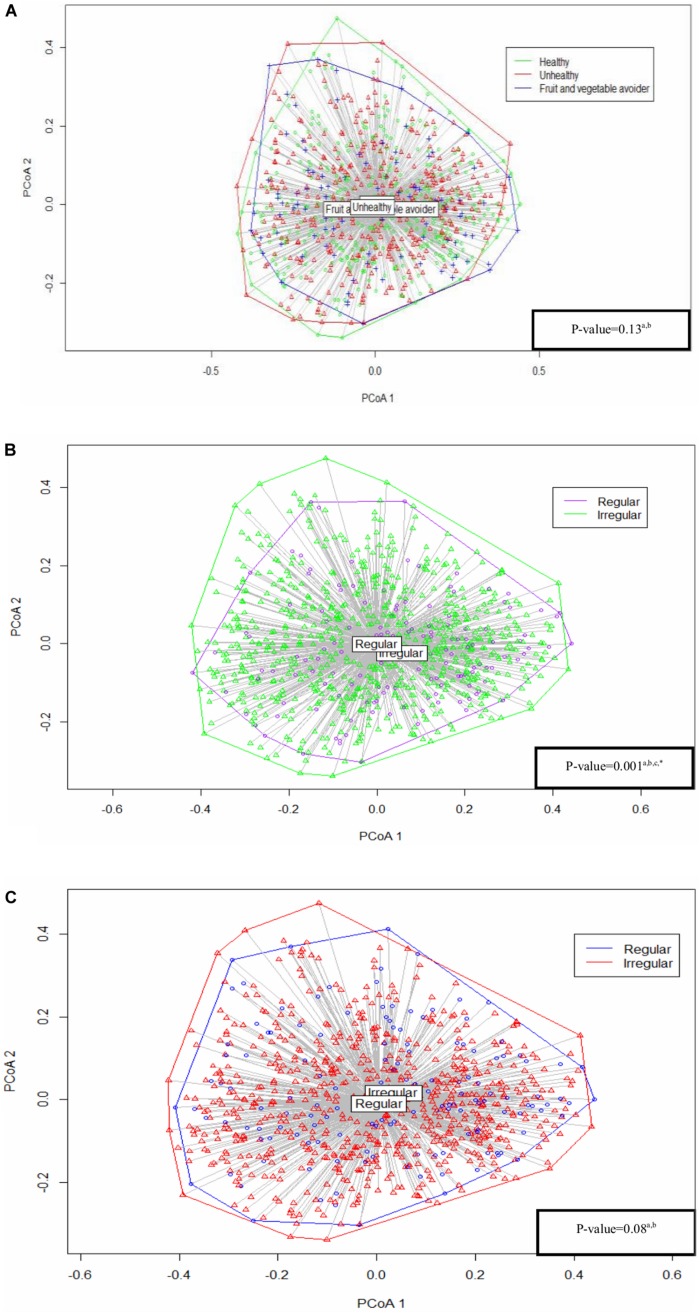
Analysis based on Bray–Curtis dissimilarity between **(A)** eating habits, **(B)** breakfast patterns, and **(C)** dinner patterns. All the *p*-values were adjusted and tested by the permutational analysis of variance (PERMANOVA) test. ^a^Adjusted for: gender, age, parental language, body mass index (BMI) categories and sequencing depth. ^b^Results from permutational analysis of variance (PERMANOVA) test. ^c^Unbalanced design. *Statistically significant.

The Bray–Curtis index assessed the dissimilarity of saliva microbiota between eating habits. There was no significant difference between eating habits (PERMANOVA: *p* = 0.13) (Figure 2). Similarly, sensitivity analyses showed no statistical significance between eating habits in alpha diversity and bacterial composition in the regular breakfast and dinner eaters’ subgroups (Supplementary Table S2).

Differences in abundance for OTUs between eating habits are shown in Table 2 and Supplementary Figure S2. Six OTUs were higher abundant in FV avoiders compared with healthy eaters. A higher abundance was found in one unclassified bacteria belonging to the Candidate division SR1 phylum and one unclassified bacterium belonging to the *Neisseriaceae* family in FV avoiders compared with healthy eaters (Table 2). Additionally, we found high abundances of *Prevotella* (Otu003 and Otu023), *Dialister* and one unclassified bacterium belonging to the *Prevotellaceae* family (Otu213). Unhealthy eaters had a lower abundance of an unclassified bacterium belonging to the *Lachnospiraceae* family (Table 2) compared with the healthy group. In turn, *Megasphaera* was more abundant in the unhealthy group compared with the healthy group. There were no differences in OTU abundances between FV avoiders and unhealthy eaters.

**TABLE 2:** Differentially abundant bacteria of adolescents by eating habits and breakfast and dinner patterns.

	Group	OTU	Nearest taxa	Base mean OTU	Log2Fold change	Log2Fold SE	*p*-value	Adjusted *p*-value
Eating habit groups^a^	Fruit and vegetable avoider vs. Healthy	*Otu*171	Unclassified bacteria from Neisseriaceae family	3.20	2.44	0.58	2.74E−05	0.006
		*Otu*003	Prevotella	2370.05	0.38	0.10	7.28E−05	0.006
		*Otu*023	Prevotella	315.48	0.47	0.12	8.59E−05	0.006
		Otu106	Unclassified bacteria from Candidate division SR1 phylum	11.72	0.71	0.21	0.001	0.036
		*Otu*213	Unclassified bacteria from Prevotellaceae family	1.20	1.43	0.42	0.001	0.036
		*Otu*113	Dialister	8.52	0.54	0.17	0.001	0.042
	Unhealthy vs. Healthy	*Otu*587	Unclassified bacteria from Lachnospiraceae family	0.05	–23.00	4.81	1.75E−06	0.003
		*Otu*280	Megasphaere	0.90	2.20	0.48	4.75E−06	0.004
	Fruit and vegetable avoider vs. Unhealthy	Non-significant						
Breakfast patterns^a^	Irregular vs. Regular	*Otu*023	Prevotella	315.48	0.64	0.15	2.24E−05	0.008
		Otu027	Unclassified bacteria from Candidate division TM7 phylum	153.31	0.80	0.21	0.0002	0.027
		*Otu*128	Sphingomonas	7.47	–2.00	0.55	0.0002	0.027
		*Otu*002	Veillonella	4320.25	0.55	0.16	0.0004	0.030
		*Otu*178	Veillonella	2.10	0.63	0.18	0.0004	0.030
		*Otu*035	Derxia	101.92	–0.53	0.16	0.001	0.048
Dinner patterns^a^	Irregular vs. Regular	*Otu*063	Prevotella	39.40	1.02	0.21	1.80E−06	0.0003
		*Otu*078	Porphyromonas	23.55	–0.92	0.23	4.57E−05	0.003
		*Otu*088	Campylobacter	18.86	–0.82	0.20	3.57E−05	0.003
		*Otu*073	Capnocytophaga	28.69	–0.63	0.17	0.0003	0.013
		*Otu*039	Haemophilus	76.32	–1.15	0.33	0.001	0.019
		*Otu*052	Actinobacillus	55.06	–0.70	0.21	0.001	0.028
		*Otu*016	Porphyromonas	522.59	–0.47	0.15	0.002	0.046
		*Otu*050	Johnsonella	58.49	–0.39	0.13	0.002	0.046
		*Otu*157	Neisseria	3.11	–1.30	0.42	0.002	0.046
		*Otu*204	Leptotrichia	1.56	1.84	0.61	0.002	0.046
		*Otu*006	Neisseria	1903.29	–0.50	0.17	0.003	0.048

*^a^Adjusted for: gender, age, parental language, body mass index (BMI) categories and sequencing depth. OTU, operational taxonomic unit; Log2Fold SE, Log2Fold standard error.*

### Breakfast Patterns and Saliva Microbiota

A higher alpha diversity was shown in the regular breakfast pattern group compared with the irregular breakfast pattern group regarding the inverse Simpson’s index [6.27 (0.17) vs. 5.80 (0.02), ANCOVA: *p* = 0.01] and Shannon diversity index [2.27 (0.03) vs. 2.22 (0.03), ANCOVA: *p* = 0.06] (Figure 1). Further adjustment for eating habits retained the differences between regular and irregular breakfast eaters (Supplementary Table S1). No interaction was observed between gender and breakfast patterns using the Shannon diversity (*p* = 0.97) and inverse Simpson’s indices (*p* = 0.91).

The dissimilarity index differed between regular and irregular breakfast pattern groups (PERMANOVA: *p* = 0.001), indicating small differences in bacterial composition between the two groups (Figure 2). Results were further confirmed using a balanced design, as described in Materials and Methods section (Supplementary Figure S3). We were able to repeat our findings in the sensitivity analyses focusing entirely on healthy eaters’ subgroup, but not in FV avoiders’ subgroup (Supplementary Table S2).

We identified several OTUs that differed in abundances between regular and irregular breakfast eaters (Supplementary Figure S2 and Table 2). Irregular breakfast eaters had a lower abundance of *Sphingomonas* compared with the regular breakfast eaters (Table 2). Four bacteria were more abundant in the irregular breakfast pattern group compared with the regular breakfast pattern group. A higher abundance was observed in one unclassified bacterium belonging to the Candidate division TM7 phylum in the irregular breakfast pattern group compared with the regular breakfast pattern group. Similar to FV avoiders, irregular breakfast eaters had a high abundance of *Prevotella* (Otu023). A higher abundance was also observed for two *Veillonella* OTUs (Otu002 and Otu178) in irregular breakfast eaters compared with regular breakfast eaters.

### Dinner Patterns and Saliva Microbiota

A higher alpha diversity was identified in the regular dinner pattern group compared with the irregular dinner pattern group using the Shannon diversity index [2.27 (0.03) vs. 2.22 (0.03), ANCOVA: *p* = 0.054] but not with inverse Simpson’s index [6.23 (0.17) vs. 6.04 (0.22), ANCOVA: *p* = 0.28] (Figure 1). Results were further adjusted for eating habits showing similarly differences between regular and irregular dinner eaters with the Shannon diversity index but not with inverse Simpson’s index (Supplementary Table S1). Moreover, there was no interaction between gender and dinner patterns related to the alpha diversity (Shannon diversity index *p* = 0.85, inverse Simpson’s index *p* = 0.96). In the FV avoiders’ subgroup, we confirmed our findings on dinner eating in the sensitivity analyses but not in healthy eaters’ subgroup (Supplementary Table S2).

Regular and irregular dinner eaters showed no difference in composition (PERMANOVA: *p* = 0.08) (Figure 2). Similar findings were found in dinner eating in healthy eaters’ subgroup, while bacterial composition differed between regular and irregular dinner eaters in FV avoiders’ subgroup in the sensitivity analyses (Supplementary Table S2).

We identified bacteria that differed in abundance between regular and irregular dinner eaters’s groups (Supplementary Figure S2 and Table 2). Similarly to FV avoiders and irregular breakfast eaters, irregular dinner eaters showed a high abundance of *Prevotella*, but in a different OTU (Otu063) (Table 2). Additionally, irregular dinner eaters had a high abundance of *Leptotrichia*. However, several bacteria were less abundant in irregular eaters. Specifically, lower abundance of *Neisseria* and *Haemophilus* were found in irregular dinner eaters compared with regular dinner eaters.

## Discussion

This study including 842 adolescents found no differences between saliva microbiota diversity and composition between healthy, unhealthy and FV avoiders. However, adolescents who ate breakfast regularly had a higher diversity compared with irregular breakfast eaters. Additionally, regular and irregular breakfast eaters differed slightly in saliva microbiota composition. To support this, a higher diversity was observed correspondingly in regular dinner eaters. We identified several bacteria that differed in abundances between eating habits, between irregular and regular breakfast eaters, and between irregular and regular dinner eaters. These associations reflect a combination of habitual and actual eating patterns since the timing of the saliva sample donation from last meal was unknown.

This is the first paper to report the associations between eating habits and saliva microbiota in adolescents. Moreover, very little is known about associations between eating habits and saliva microbiota at any age. A somewhat similar study (De Filippis et al., 2014) on saliva microbiota did not identify differences between omnivores, non-omnivore diets, supporting our findings. However, our study differed notably from earlier related diet and saliva microbiota studies (De Filippis et al., 2014; Ercolini et al., 2015; Hansen et al., 2018) as we did not examine any strictly followed diet, therefore, previous studies are not directly comparable to our findings. Additionally, the previous studies used either 4-day weighted food record (Hansen et al., 2018) or a daily food diary before and during the sampling (De Filippis et al., 2014). While we used a 14-item FFQ to receive information on indicatory food items and describe the eating habits based on these. Previously, saliva microbiota has shown to remain stable toward alterations in diet (Stahringer et al., 2012).

To our knowledge, there are no earlier studies on the association of breakfast and dinner patterns with the saliva microbiota. Most similar to our study is a cross-over study in women which observed that eating the main meal late causes changes in the daily rhythm of alpha diversity in saliva, which may have an effect of host’s metabolism (Collado et al., 2018). Based on our findings, we suggest that our irregular eaters may have a higher risk of diversity loss than regular eaters.

Previously, we have linked irregular breakfast to excess weight in Finnish adolescents (Viljakainen et al., 2019). There is strong evidence of an association between an irregular breakfast pattern and overweight and obesity in European children and adolescents (Szajewska and Ruszczynski, 2010; Viljakainen et al., 2019). While following a regular meal pattern was linked to a low risk of overweight and obesity in Finnish adolescents (Jääskeläinen et al., 2013). In fact, we have previously reported a lower diversity in overweight and obese adolescents than normal weight adolescents in the same material (Raju et al., 2019). Moreover, we found associations of regular breakfast and dinner eaters with a higher diversity and more dissimilar composition in the saliva microbiota than in irregular eaters. Therefore, our findings are unique and may provide insight into why regular eating promotes normal-weight. Generally, more diverse ecosystems are beneficial for human microbiota (Levine and D’Antonio, 1999) and our results support this.

In the present study, FV avoiders and irregular breakfast and dinner patterns had a high abundance of *Prevotella*. In the saliva, *Prevotella* has been shown to be less abundant in vegans than omnivores (Hansen et al., 2018). Oral *Prevotella*, such as *P. intermedia, P. oralis*, and *P. gingivalis* (Nadkarni et al., 2012) are known for degrading proteins and peptides into amino acids (Takahashi, 2015). Moreover, a high abundance of *Prevotella* has been linked to periodontal disease (Wang et al., 2013). Gut *Prevotella*, predominantly *P. copri*, has been linked to a fiber and polysaccharide-rich diet and thus proposed to be a biomarker of both diet and lifestyle (Gorvitovskaia et al., 2016). Based on our findings, we propose that the association of FV avoiders and meal skippers with oral *Prevotella* is prompting future research on unhealthy eating habits and oral *Prevotella*.

Among irregular breakfast eaters, Candidate division TM7 had a higher abundance than in regular breakfast eaters. A high abundance of TM7 rDNA has been discovered in patients with mild periodontitis (Brinig et al., 2003). In addition to Candidate division TM7, *Veillonella* was highly abundant in irregular breakfast eaters. A previous study (Mashima et al., 2017) on salivary microbiota and oral hygiene found that *Veillonella* typically presents in children with poor oral hygiene. *Veillonella* is also known for utilizing carbohydrates, such as glucose, or amino acids for energy mobilization (Delwiche et al., 1985). In our earlier study, irregular breakfast eaters more often had unhealthy eating habits, including preferring sugary juice drinks and sweets and avoiding fruits and vegetables, than regular breakfast eaters (de Oliveira Figueiredo et al., 2019). Unfortunately, data on the oral health of the adolescents was not available. Oral health has been reported to be at satisfactory levels in Finnish adolescents and more than a quarter of 12-year-olds were caries-free in 2009 (Widstrom and Järvinen, 2011). The large majority of Finnish children do not belong to the risk group for periodontitis since it is more common in adults and the prevalence increases with age (Suominen-Taipale et al., 2008). However, our findings need to be confirmed using oral health status data. Generally, a regular meal pattern is an indicator of healthy eating. Thus, regularity of meals can influence on health outcomes, such as protecting from overweight and obesity in adolescents (Jääskeläinen et al., 2013) and may contribute positively to oral health as well.

We identified different bacteria in irregular dinner eaters, FV avoiders and unhealthy eaters, which have been linked to caries (Eriksson et al., 2017). More specifically, irregular dinner eaters had a high abundance of *Leptotrichia*, which is known for the production of an acidic environment (Hofstad, 1992). *Leptotrichia* is reliant on tooth eruption (Hofstad, 1992). Caries are caused by bacteria producing acid from metabolized sugars (World Health Organization [WHO], 2017). Earlier studies reported that *Neisseria* (Hurley et al., 2019) and *Megasphaere* (Xu et al., 2018) have also been enriched in children with caries. In our study, both *Neisseria* and *Megaspaere* were highly abundant in FV avoiders and in unhealthy eaters. One of the reasons behind the development of caries is frequent consumption of sugar-containing foods, such as sugary juice drinks and sweets, which include high amounts of free sugars (World Health Organization [WHO], 2017) and are favored by adolescents (Diethelm et al., 2012) over fruits and vegetables (Hoppu et al., 2010). Our earlier study (de Oliveira Figueiredo et al., 2019) observed that adolescents who mostly followed irregular meal patterns (regarding lunch and dinner) were also unhealthy eaters. Another unhealthy way of eating is avoiding fruits and vegetables. Thus, irregular dinner eaters, unhealthy eaters, and FV avoiders may be at a high risk of poor oral health based on the abundance of microbes.

A strength of our study is the large sample size. This large study permitted us to examine saliva microbiota by eating habits and breakfast and dinner consumption. Our study advances our understanding of healthy eating, particularly how vital the regularity of meals is to the diversity and composition of our saliva microbiota. Future studies are also needed to examine association between different dietary patterns and saliva microbiota. Study limitations include the missing information on oral health which is a possible confounder for our findings, and our brief non-validated food frequency questionnaire providing data only on indicatory food items. Therefore, we do not have information on portion size and energy intake. We also need more details on other food items that the food frequency questionnaire did not record among FV avoiders and irregular breakfast and dinner eaters. Furthermore, we could not separate habitual from actual eating habits on the day of sample donation, since the cohort lacks information on the timing of last meal. Therefore, skipping breakfast on the day of sampling could not be accounted for. We could not standardize for meal behaviors prior to saliva sampling in this age group without severely influencing participation rates. However, we conducted sensitivity analyses in eating habits, breakfast and dinner patterns to reduce the variability by stratification. However, associations of habitual meal patterns are probable since we identified associations with dinner patterns in samples collected prior to dinner. DEseq2 may report false positive differences in relative abundances since an increase in one OTU will result reductions in the remaining OTUs. However, this effect is neglectable in rich salivary communities.

## Conclusion

Meal regularity plays a larger role in shaping the saliva microbiota than eating habits. Regularity of breakfast eating was associated with both saliva diversity and composition: regular breakfast eaters had a higher diversity compared with irregular breakfast eaters. A higher diversity was also observed in regular dinner eaters compared with irregular dinner eaters. *Prevotella* abundances in saliva may be an indicatory bacterium for unhealthy eating, however this warrants further research.

## Data Availability Statement

The datasets generated for this study can be found in the EGA database (Accession number EGAS00001003039).

## Ethics Statements

This study was carried out in accordance with the recommendations of regional Ethics Committee of the Hospital District of Helsinki and Uusimaa. The protocol was approved by the regional Ethics Committee of the Hospital District of Helsinki and Uusimaa (169/13/03/00/10). All subjects gave written informed consent in accordance with the Declaration of Helsinki.

## Author Contributions

JV, SR, HV, RF, and TR conducted the statistical analyses. JV was responsible for drafting the manuscript. All authors contributed to the study design and planning, interpreted the results, revised and approved the manuscript.

## Disclaimer

Where authors are identified as personnel of the International Agency for Research on Cancer/World Health Organization, the authors alone are responsible for the views expressed in this article and they do not necessarily represent the decisions, policy or views of the International Agency for Research on Cancer/World Health Organization.

## Conflict of Interest

The authors declare that the research was conducted in the absence of any commercial or financial relationships that could be construed as a potential conflict of interest.
