# Supplementary material for: Meal Regularity Plays a Role in Shaping the Saliva Microbiota
Source: Front Microbiol. 2020 Apr 24;11:757. doi: 10.3389/fmicb.2020.00757 (PMC7194025; doi:10.3389/fmicb.2020.00757)
Supplement: Supplementary file 1 [file Data_Sheet_1.PDF]

Supplementary Material

Meal regularity plays a role in shaping the saliva microbiota

Jannina Viljakainen[1-2], Sajan C. Raju[1-2], Heli Viljakainen[1,3], Rejane Augusta de Oliveira Figueiredo[1-2], Eva Roos[1-3], Elisabete Weiderpass[5,a], Trine B Rounge[1-2,4,6*,a]

[1]Folkhälsan Research Center, Helsinki, Finland

[2]Faculty of Medicine, University of Helsinki, Helsinki, Finland

[3]Department of Food and Nutrition, University of Helsinki, Helsinki, Finland

[4]Department of Research, Cancer Registry of Norway, Oslo, Norway

[5]International Agency for Research on Cancer – World Health Organization, Lyon, France

[6]Department of Informatics, University of Oslo, Norway

[a]Equal contribution

1.1 Supplementary Figures

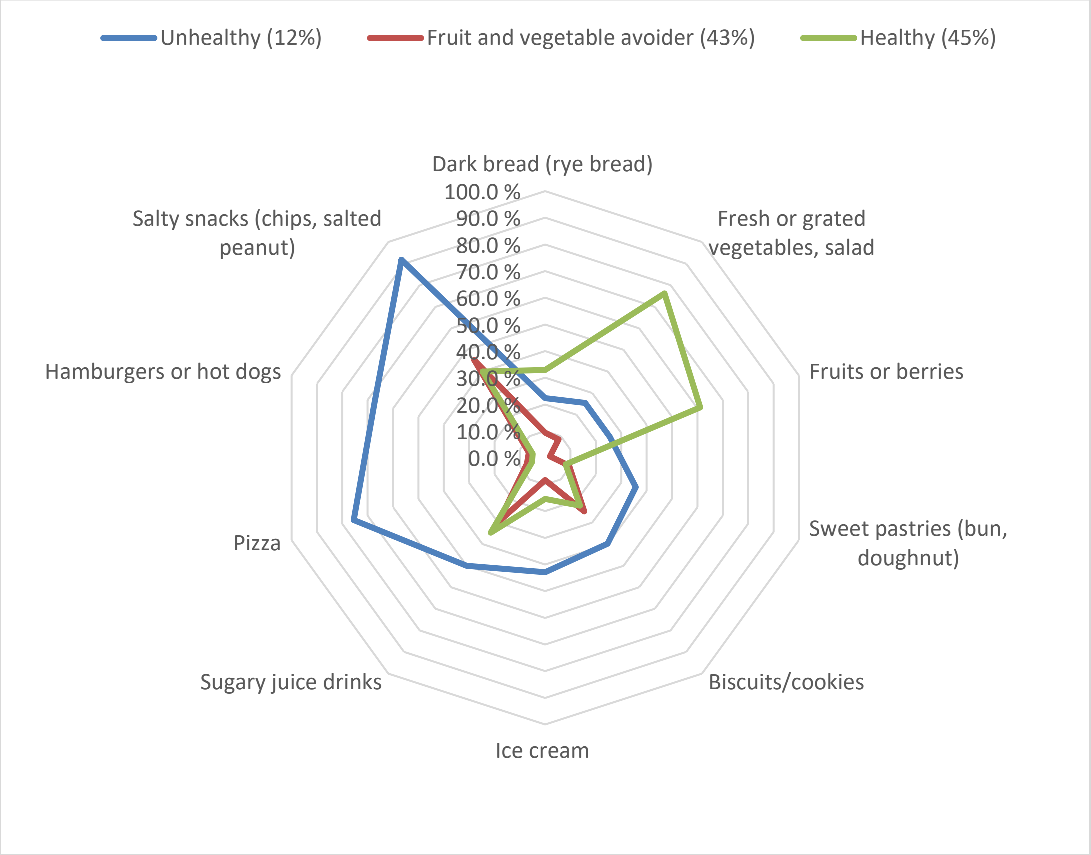

Supplementary Figure 1. Frequency of high consumption of specified food items by unhealthy eaters (unhealthy), FV avoiders (fruit and vegetable avoider) and healthy eaters (healthy).

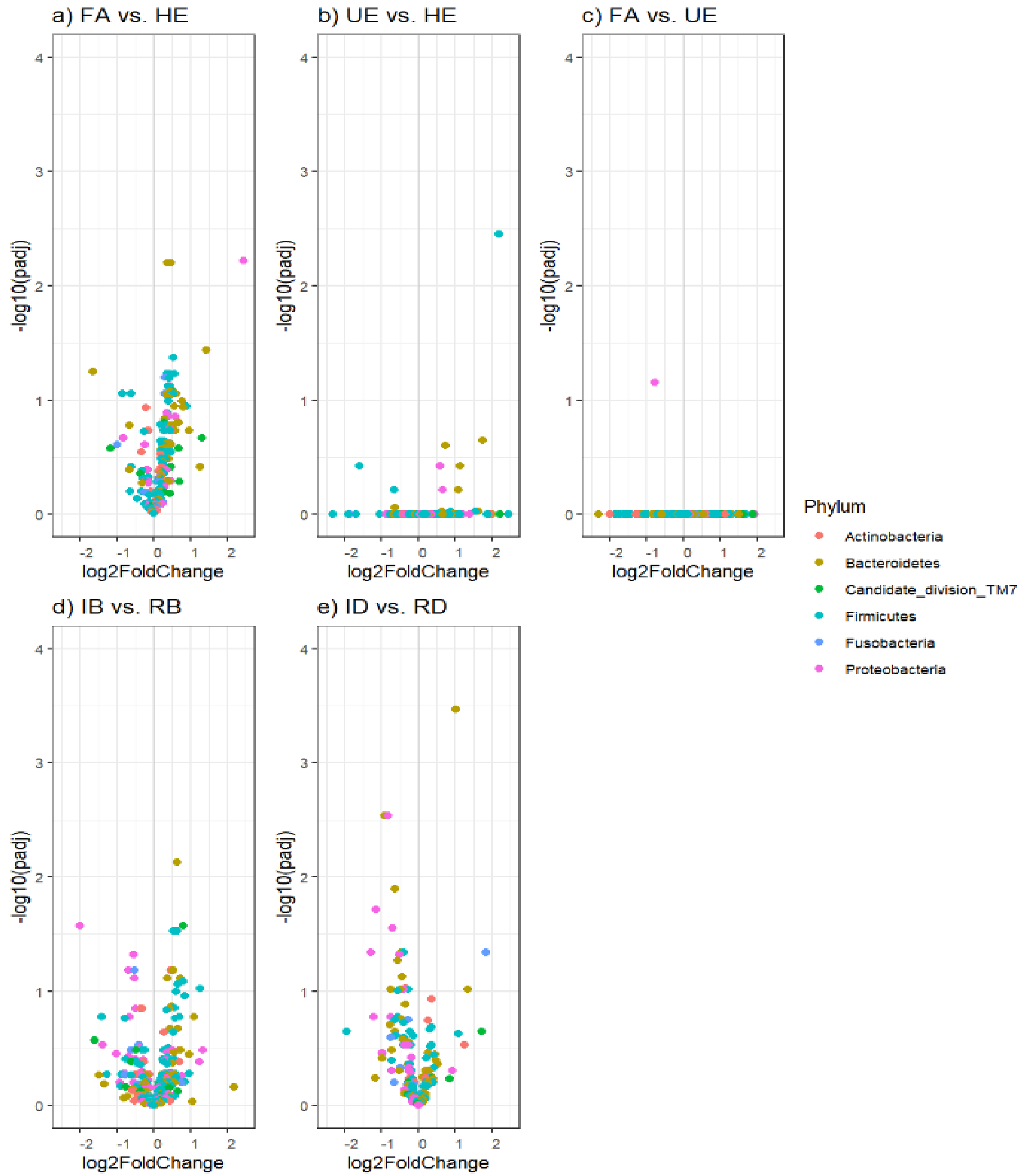

Supplementary Figure 2. Differentially abundant microbes in saliva between a) FV avoiders vs. healthy (FA vs. HE), b) unhealthy vs. healthy (UE vs. HE), c) FV avoiders vs. unhealthy (FA vs. UE), d) irregular breakfast vs. regular breakfast patterns (IB vs. RB), and e) irregular dinner vs. regular dinner patterns (ID vs. RD). Differentially abundant OTUs were colored based on their assigned phyla.

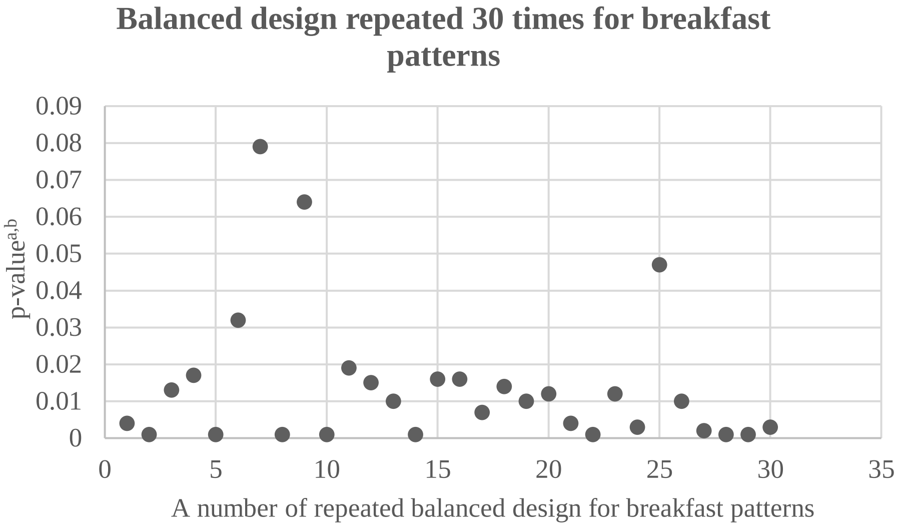

Supplementary Figure 3. Bray-Curtis dissimilarity between breakfast patterns. [a]Adjusted for: gender, age, parental language, body mass index (BMI) categories and sequencing depth. [b]Results for permutational analysis of variance (PERMANOVA) test.

1.2 Supplementary Tables

Table 1S. Alpha diversity and Bray-Curtis dissimilarity between breakfast and dinner patterns.

	Shannon index		Inverse Simpson's index		Bray-Curtis dissimilarity index
	Mean (SEM)	p-value[1,2]	Mean (SEM)	p-value[1,2]	p-value[1,3]
Breakfast patterns					
Regular (n=700)	2.27 (0.03)	0.040	6.29 (0.18)	0.007	0.001
Irregular (n=142)	2.22 (0.03)		5.79 (0.23)		
Dinner patterns					
Regular (n=694)	2.27 (0.03)	0.042	6.24 (0.18)	0.248	0.080
Irregular (n=148)	2.22 (0.03)		6.03 (0.22)		

[1]Adjusted for: eating habits, gender, age, parental language, body mass index (BMI) categories and sequencing depth.[2]All the adjusted p-values were calculated with Analysis of Covariance (ANCOVA).[c]Results from permutational analysis of variance (PERMANOVA) test. SEM=Standard Error of Means.

Table 2S. Sensitivity analyses for eating habits and breakfast and dinner patterns.

	Shannon index		Inverse Simpson's index		Bray-Curtis dissimilarity index
	Mean (SEM)	p-value[1,2]	Mean (SEM)	p-value[1,2]	p-value[1,3]
Regular breakfast eaters					
Eating habits					
Healthy (n=344)	2.24 (0.03)		6.13 (0.21)		
Unhealthy (n=72)	2.27 (0.04)	0.568	6.29 (0.29)	0.811	0.300
Fruit and vegetable avoider (n=284)	2.26 (0.03)		6.18 (0.21)		
Regular dinner eaters					
Eating habits					
Healthy (n=338)	2.24 (0.03)		6.06 (0.21)		
Unhealthy (n=74)	2.26 (0.04)	0.319	6.20 (0.28)	0.658	0.337
Fruit and vegetable avoider (n=282)	2.28 (0.03)		6.19 (0.21)		
Healthy eaters					
Breakfast patterns					
Regular (n=344)	2.27 (0.04)	0.340	6.37 (0.26)	0.023	0.017
Irregular (n=39)	2.22 (0.06)		5.59 (0.39)		
Dinner patterns					
Regular (n=338)	2.27 (0.04)	0.924	6.24 (0.26)	0.822	0.176
Irregular (n=45)	2.26 (0.06)		6.31 (0.37)		
Fruit and vegetable avoiders					
Breakfast patterns					
Regular(n=284)	2.24 (0.04)	0.164	6.10 (0.25)	0.215	0.174
Irregular(n=77)	2.19 (0.05)		5.79 (0.33)		
Dinner patterns					
Regular (n=282)	2.26 (0.04)	0.016	6.15 (0.26)	0.125	0.028
Irregular (n=79)	2.17 (0.05)		5.78 (0.31)		

[1]Adjusted for: gender, age, parental language, body mass index (BMI) categories and sequencing depth.[2]All the adjusted p-values were calculated with Analysis of Covariance (ANCOVA).[c]Results from permutational analysis of variance (PERMANOVA) test. SEM=Standard Error of Means.
